# Harnessing routine MRI for the early screening of Parkinson’s disease: a multicenter machine learning study using T2-weighted FLAIR imaging

**DOI:** 10.1186/s13244-025-01961-3

**Published:** 2025-04-26

**Authors:** Junyan Fu, Hongyi Chen, Chengling Xu, Zhongzheng Jia, Qingqing Lu, Haiyan Zhang, Yue Hu, Kun Lv, Jun Zhang, Daoying Geng

**Affiliations:** 1 Department of Radiology, Huashan Hospital, Fudan University, Shanghai, China; 2 Academy for Engineering and Technology, Fudan University, Shanghai, China; 3 Department of Radiology, Affiliated Hospital of Nantong University, Nantong, China; 4 Department of Radiology, The First Affiliated Hospital of Ningbo University, Ningbo, China; 5 Department of Radiology, The Second Affiliated Hospital of Xuzhou Medical University, Xuzhou, China; 6 Center for Shanghai Intelligent Imaging for Critical Brain Diseases Engineering and Technology Research, Shanghai, China; 7 Institute of Functional and Molecular Medical Imaging, Fudan University, Shanghai, China

**Keywords:** Parkinson’s disease, Machine learning, Magnetic resonance imaging, Substantia nigra, Putamen

## Abstract

**Objective:**

To explore the potential of radiomics features derived from T2-weighted fluid-attenuated inversion recovery (T2W FLAIR) images to distinguish idiopathic Parkinson’s disease (PD) patients from healthy controls (HCs).

**Methods:**

T2W FLAIR images from 1727 subjects were retrospectively obtained from five cohorts and divided into a training set (395 PD/574 HC), an internal test set (99 PD/144 HC) and an external test set (295 PD/220 HC). Regions of interest (ROIs), including bilateral globus pallidus (GP), putamen (PU), substantia nigra (SN), and red nucleus (RN), were manually delineated. The radiomics features were extracted from ROIs. Six independent machine learning (ML) classifiers were trained on the training set, and validated on the internal and external test sets.

**Results:**

A selection of five, two, three, and ten highly correlated diagnostic features were identified from SN, RN, GP, and PU regions, respectively. Six ML classifiers were implemented based on the combined 20 radiomics features. In the internal test cohort, the six models achieved AUC of 0.96–0.98 with the accuracy ranging from 0.80 to 0.90. In the external test cohort, the multilayer perceptron model demonstrated the highest AUC of 0.85 (95% CI: 0.80–0.89) with an accuracy of 0.78.

**Conclusion:**

ML models based on the conventional T2W FLAIR images demonstrated promising diagnostic performance for PD and those models may serve as a basis for future investigations on PD diagnosis with the aid of ML methods.

**Critical relevance statement:**

Our study confirmed that early screening of Parkinson’s Disease based on the conventional T2W FLAIR images was feasible with the aid of machine learning algorithms in a large multicenter cohort and those models had certain generalization.

**Key Points:**

- Conventional head MRI is routinely performed in Parkinson’s disease (PD) but exhibits inadequate specificity for diagnosis.
- Machine learning (ML) models based on conventional T2W FLAIR images showed favorable accuracy for PD diagnosis.
- ML algorithm enables early screening of PD on routine T2W FLAIR sequence.

**Graphical Abstract:**

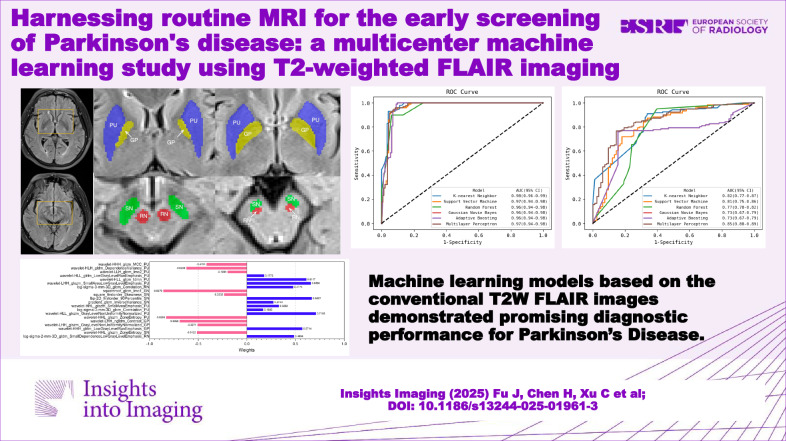

## Introduction

Parkinson’s disease (PD) stands as the second most prevalent neurodegenerative disorder, with an estimated six million individuals affected globally in 2016 [1]. It is characterized by tremor, rigidity, bradykinesia, or postural instability accompanied by a series of non-motor symptoms such as autonomic dysfunction, olfactory loss, and rapid eye movement sleep behavior disorder [1]. The aberrant α-synuclein aggregation and loss of dopaminergic neurons in the substantia nigra (SN) and striatum have been elucidated as the main underlying pathophysiology of PD and to be irreversible. By the time motor symptoms become apparent, there may be a loss of 40–60% of nigral dopaminergic neurons and up to an 80% reduction in synaptic function [2, 3]. Therefore, early screening of PD is critical for timely intervention and potential slowing of disease progression.

In daily practice, the diagnosis of PD heavily relies on history taking, neurological examination, and a response to dopaminergic therapy; conventional head MRI is routinely performed to exclude secondary pathology in PD but lacks specificity for diagnosis [4]. In this current mode, diagnostic errors are common due to the heterogeneity of clinical manifestations and extended latency of PD. A meta-analysis showed that this misclassification rate of PD could reach 16.1% at first visit and 20.4% in the follow-up [5]. Only 53% accuracy was reported in PD with less than 5 years of disease duration [6]. Imaging examinations including dopamine-SPECT and ^18^F-FDG PET imaging have proven valuable for PD diagnosis [7]. However, few hospitals are currently able to carry out the relevant technology due to the high cost and high specificity of radiotracers. Advanced MRI techniques, such as quantitative susceptibility mapping, diffusion MRI and functional MRI, have unveiled midbrain and neostriatum abnormalities in PD from various pathophysiological perspectives and performed certain guiding value in the diagnosis of PD [8–11]. Unfortunately, those analyses are complex and rely on the experience of senior neuroradiologists, which hinders their clinical application. Therefore, it is highly desirable to develop accessible, non-invasive, and low-cost biomarkers to improve the accuracy of PD diagnosis.

Recently, the advent of computer-aided diagnosis, leveraging machine learning (ML), has shown promise in improving the diagnostic yield of MRI. It can provide the intrinsic details and heterogeneous properties of tissues, thereby detecting subtle changes within an area of interest [12]. ML has also shown great potential in extracting radiomics features on MRI to effectively distinguish PD. Our previous study has extracted radiomics features from SN in the neuromelanin-sensitive MRI and the best-performing ML model reported an accuracy of 0.889 and 0.875 in the test and external validation sets, respectively [13]. More precise differentiation between PD and other forms of parkinsonism has been achieved with the aid of ML based on susceptibility-weighted imaging data [14, 15] and 3D T1-weighted MRI [16], even in the absence of visually discernible changes in brain subregions. Nevertheless, the radiomics model of PD diagnosis based on conventional MRI has not yet been developed. There has been a small-scale study that utilized the radiomic features of neostriatum from conventional T2-weighted images to distinguish PD from healthy controls (HCs), which achieved an area under the receiver operating characteristic curve (AUC) over 0.70 both in the training and test groups [17]. This result suggested that the conventional MRI combing radiomics approach may play a gain role in PD diagnosis, but further investigation is needed in a larger cohort.

This study hypothesized that ML could serve as a powerful adjunct to conventional MRI for the early identification of PD, providing intelligent decision support for clinical diagnosis. We aimed to investigate the feasibility of radiomics features extracted from the routinely acquired T2-weighted fluid-attenuated inversion recovery (T2W FLAIR) images in classifying PD from HCs.

## Materials and methods

### Participants

**Fig. 1:**
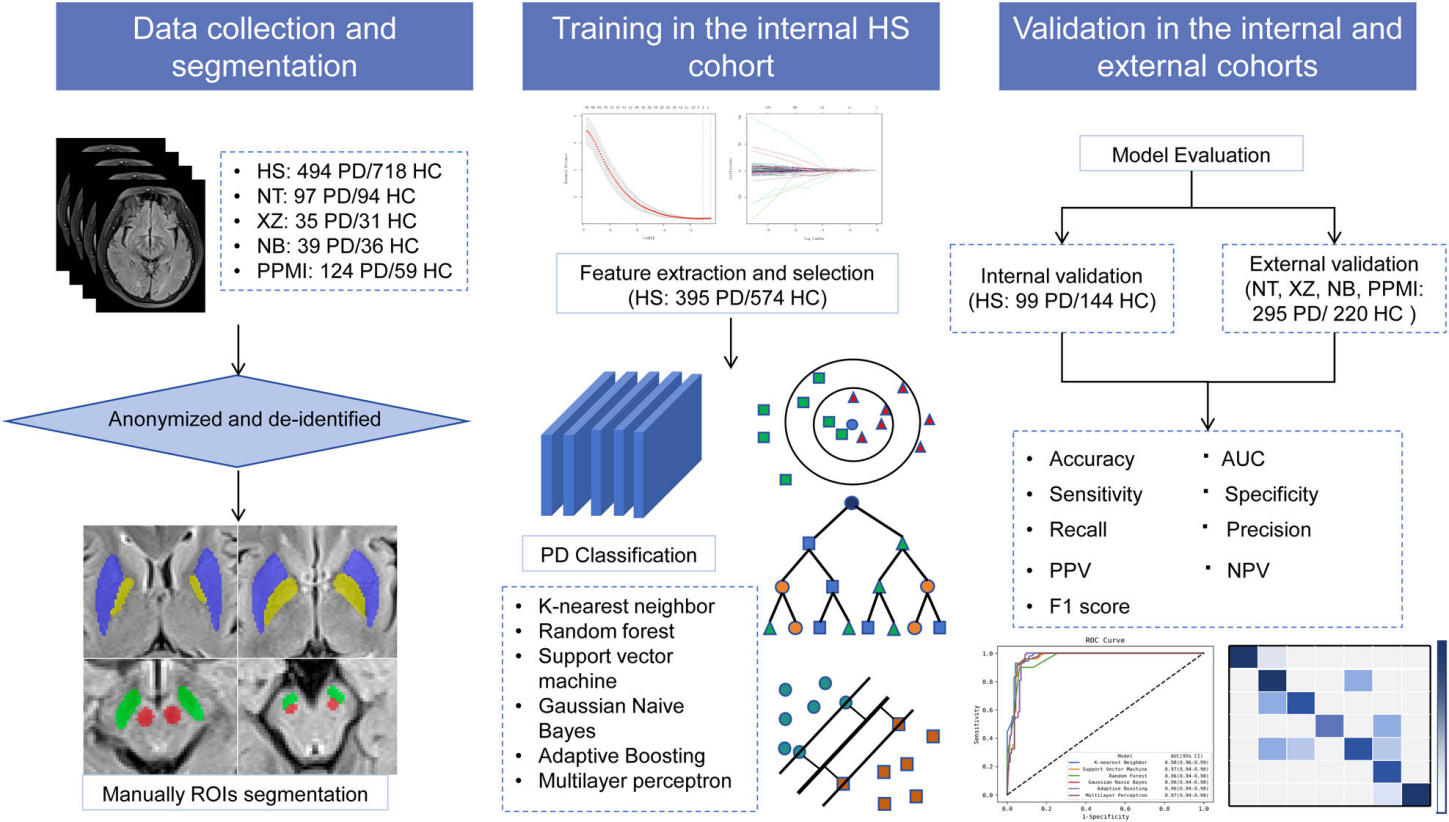
Workflow of classification models for PD. PD, Parkinson’s disease; HC, healthy control; HS, Huashan Hospital affiliated to Fudan University; NT, Affiliated Hospital of Nantong University; XZ, The Second Affiliated Hospital of Xuzhou Medical University; NB, Ningbo First Hospital; PPMI, Parkinson’s Progression Marker Initiative; AUC, area under the receiver operating characteristic curve; PPV, positive predictive value; NPV, negative predictive value

Our investigation encompassed a cohort of 1727 subjects, recruited from four hospitals and the Parkinson’s Progression Marker Initiative (PPMI, https://www.ppmi-info.org). This retrospective study was approved by the local Ethics Committees at Huashan Hospital (KY2022-032), and the written informed consent was waived by the Ethics Committees. The workflow of this study is demonstrated in Fig. 1.

### Internal training and test cohorts

All subjects in the internal cohort were enrolled from Huashan Hospital (Fudan University, Shanghai, China) between January 2019 and October 2023, defined as HS cohort. All PD patients in the HS cohort were collected from the inpatient system of the hospital, and HC subjects were collected from the physical examination unit. Subjects in the HS cohort were randomly allocated into a training cohort (395 PD/574 HC) and an internal test cohort (99 PD/144 HC) in an 8:2 ratio using the caret R package (The R Foundation for Statistical Computing, http://www.R-project.org).

### External test cohort

It incorporated datasets from the affiliated hospital of Nantong University (97 PD/94 HC), the second affiliated hospital of Xuzhou Medical University (35 PD/31 HC), Ningbo First Hospital (39 PD/36 HC) and PPMI (124 PD/59 HC). Collectively, the external test cohort comprised 295 PD patients and 220 HCs.

The inclusion criteria for PD patients were based on a clinical diagnosis of idiopathic PD according to the UK Parkinson Society Brain Bank criteria [18] or 2015 MDS clinical diagnostic criteria [19]. Exclusion criteria for PD patients were as follows: (1) a history of other neurological diseases, including cerebrovascular disease, brain tumor, head trauma, severe small cerebral vascular disease (Fazekas grade ≥ 2); (2) any other type of psychiatric disorders; (3) secondary or atypical parkinsonism; (4) unsatisfactory image quality. The HCs with age and sex matched to the PD patients were collected from the physical examination unit of the hospitals or PPMI. The inclusion criteria were as follows: (1) no neurological or psychiatric disorders; (2) older than 45 years of age; (4) satisfactory image quality without artifacts.

All participants underwent conventional head MRI including T2W FLAIR sequence. MRI and clinical data were collected from medical records of hospitals and PPMI.

### Imaging acquisition and preparation

MR images were acquired on 11 different MRI scanners, comprising 9 scanners of 3.0 T and 2 scanners of 1.5 T across five cohorts. The standard two-dimensional T2W FLAIR images used for analysis were all obtained in the axial plane, with varying slice and pixel spacing, ranging from 0.45 to 0.94 mm. The details of the imaging parameters for each cohort are provided in Supplementary Table 1.

To standardize the acquisition parameters across various MRI scanners, a series of preprocessing steps were undertaken before further segmentation. This included resampling all T2W FLAIR images to a uniform pixel spacing of 1 × 1 × 1 mm, cropping to a central region of 224 × 224 × 16 voxels using a linear interpolation method. Grayscale values were normalized to a range of [0, 255].

### Segmentation of regions-of-interest (ROIs)

**Fig. 2:**
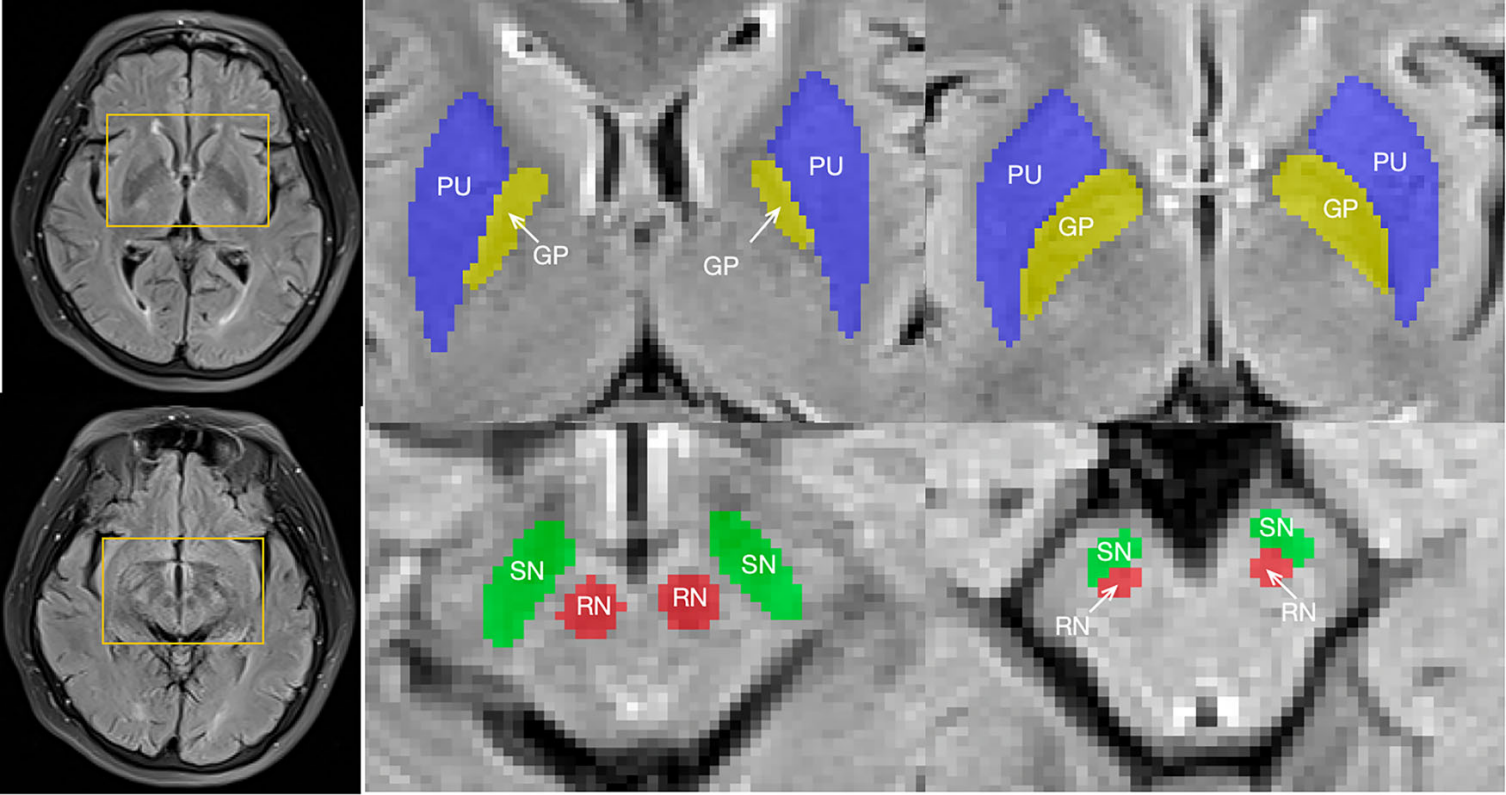
Schematic diagram of ROIs segmentation. ROIs, regions-of-interest; SN, substantia nigra; RN, red nucleus; GP, globus pallidus; PU, putamen

Drawing on existing literature, the SN, red nucleus (RN), globus pallidus (GP), and putamen (PU) are identified as key regions influenced in PD [20–23], which are well-visualized on T2W FLAIR images. Therefore, we segmented those four nuclei regions on T2W FLAIR images, and T1W, T2W and diffusion-weighted imaging sequences were utilized as the anatomical references. A schematic diagram of ROIs segmentation is shown on Fig. 2.

All data were anonymized and de-identified prior to analysis. First, two neuroradiologists with 5 and 10 years’ experience (neuroradiologist1, H.Y.; neuroradiologist2, L.K.), blinded to subjects’ state, performed the initial manual segmentation of bilateral SN, RN, GP, and PU used ITK-SNAP software (Version 3.8.0, http://www.itksnap.org). The spatial overlap of each pair of ROIs segmented by two in fifty randomized cases was compared using the Dice similarity coefficient (DSC). Then, a senior neuroradiologist (G.D.) with 20 years of experience thoroughly reviewed and refined the ROIs by the neuroradiologist1, which were then used as the final segmentation results for the following analysis.

### Radiomics feature extraction and selection

Radiomics features extraction and selection were performed with Pyradiomics (https://pyradiomics.readthedocs.io/en/latest/). Six filters were applied, including wavelet, LoG, square, square root, Local Binary Pattern in 3D (LBP3D), and Gradient. From each ROI, 1781 texture features were extracted. The extracted radiomics features included 342 first-order statistical features, 14 shape-based features, and 1425 texture features. Then, to mitigate the risk of overfitting and enhance model generalizability, we applied feature dimensionality reduction techniques. The Least Absolute Shrinkage and Selection Operator (LASSO) algorithm and Maximal Relevance and Minimal Redundancy (mRMR) algorithms were utilized to remove the redundant features and select highly related features with diagnosis.

### Model establishment

Data from HS cohort was randomly stratified into 80% training and 20% internal test sets. Model training was performed on the HS training set. Six ML algorithms, including K-nearest neighbor (KNN), random forest (RF), support vector machine (SVM), Gaussian Naive Bayes (GNB), Adaptive Boosting (AB), and multilayer perceptron (MLP) classifiers were implemented to explore which algorithm could reach the maximized diagnostic accuracy across the two conditions (PD and HCs) based on the selected features.

### Model assessment

The robustness and generalizability of the trained six classification models were accessed on the internal and external test cohorts. The diagnostic performance of models was assessed by the receiver operating characteristic (ROC) analysis. The predicted probabilities were used to calculate the AUC for each model. Accuracy, sensitivity, specificity, precision, recall, positive predictive value (PPV), negative predictive value (NPV), and F1 score were calculated by confusion matrix. The calculation formulas of the evaluation metrics are shown in Supplementary Table 2.

### Statistical analysis

The demographic characteristics were compared using two-sample *t*-test, chi-square test or Fisher’s exact test. ROC curves were drawn, and the AUC values were calculated to evaluate the classification ability of models. DSC values were calculated to evaluate the reproducibility of segmentation. Statistical differences in AUCs of the models were compared using the DeLong test in MedCalc Statistical Software (version 23.1.7). Radiomics feature extraction and selection, model establishment, and statistical analyses were processed by Python (version 3.8.3) or SPSS Statistics (version 23.0). Values were considered significant for *p* < 0.05.

## Results

### Clinical characteristics of participants

A total of 1727 subjects were enrolled, with 395 PD/574 HC in the training cohort, 99 PD/144 HC in the internal test cohort and 295 PD/220 HC in the internal test cohort. The age and sex distribution between HC and PD groups in the above cohorts were statistically indistinguishable. Demographic information of the participants is delineated in Table 1. The details of demographic information for each center in the external test cohort are presented in Supplementary Table 3.

**Table 1:** Demographic information of subjects included in the study

Cohort	Training cohort			Internal test cohort			External test cohort		
	HC	PD	*p*-value	HC	PD	*p*-value	HC	PD	*p*-value
Subjects	574	395	-	144	99	-	220	295	-
Age (years)^a^	61.74 (11.00)	62.45 (10.92)	0.322	60.86 (11.59)	62.69 (10.43)	0.202	62.85 (9.77)	61.82 (9.47)	0.974
Sex (M/F)	318/256	224/171	0.687	84/60	57/42	0.907	107/113	168/127	0.062
Disease duration (years)^b^	-	2.0 (1.0, 5.0)	-	-	3.0 (1.0, 5.0)	-	-	2.0 (1.5, 4.0)	-
Modified H&Y stage^b^	-	2.0 (1.0, 3.0)	-	-	2.0 (1.5, 3.0)	-	-	2.0 (1.5, 2.5)	-

*M* male, *F* female, *HC* healthy control, *PD* Parkinson’s disease

^a^ Data are represented as mean (standard deviation)

^b^ Data are represented as median (Q25, Q75)

### Radiomic features extraction and selection

**Fig. 3:**
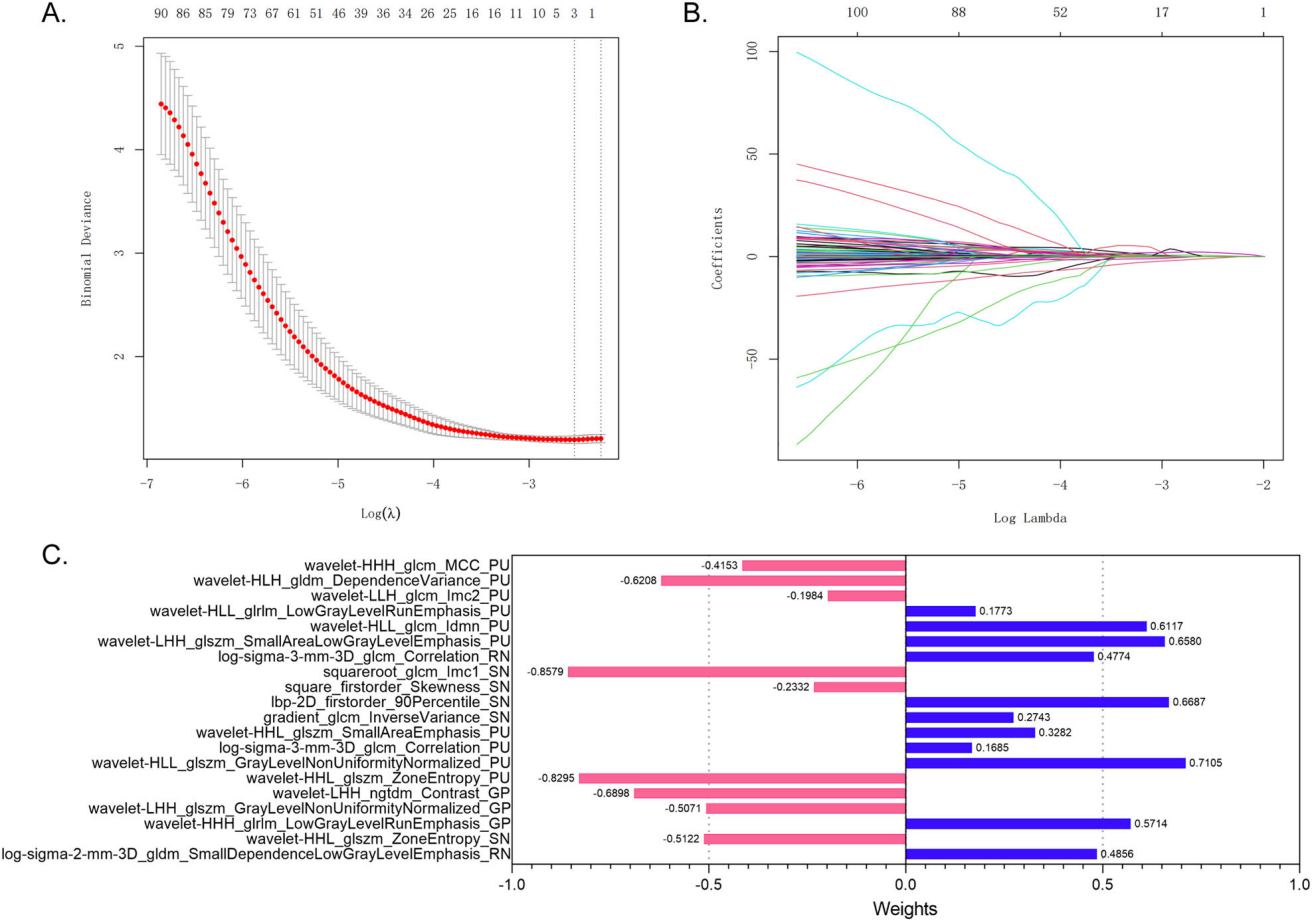
The process of dimensionality reduction and the importance of selected radiomics features. **A** The tuning parameters in the least absolute shrinkage and selection operator (LASSO) model were selected using 10-fold cross-validation. The binomial deviances were plotted vs. log (lambda) in the combined radiomics features. The left and right dotted vertical lines were drawn at the lambda in the minimum criteria and the one standard error of the minimum criteria, respectively. **B** The coefficients of radiomics features were plotted vs. log (lambda). Each colored line represented one feature. **C** The importance of the final selected 20 radiomics features generated from LASSO filter. SN, substantia nigra; RN, red nucleus; GP, globus pallidus; PU, putamen

The DSC value of ROIs segmented by two neuroradiologists was good (DSC = 0.825 ± 0.145). We extracted 1781 radiomics features from each ROI, culminating in a total of 7124 features from all ROIs. The LASSO and mRMR filters were applied and 20 features that exhibited the highest correlation with the diagnostic outcome were finally selected for the further model training process. Figure 3 depicts the process of dimensionality reduction of radiomics features and the importance of the selected radiomics features. These features, all of which were higher-order, included five from SN, two from RN, three from GP, and ten from PU. Notably, the squareroot_glcm_Imc1 feature extracted from SN demonstrated the most significant importance value of 0.8579, flowed by the wavelet-HHL_glszm_ZoneEntropy feature extracted from PU with an importance value of 0.8295.

### Internal classification performance

**Fig. 4:**
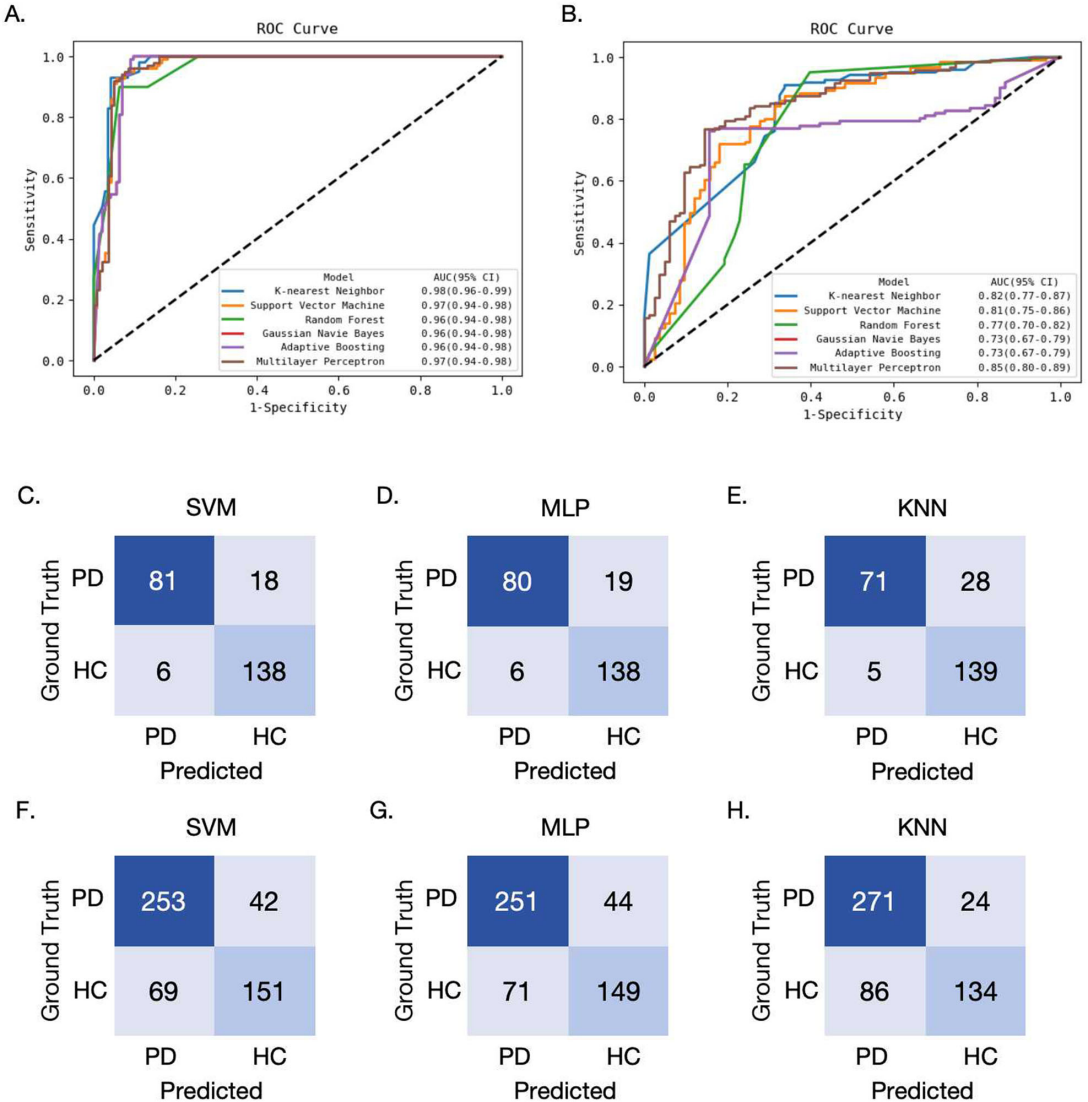
Performance of Parkinson’s disease (PD) diagnosis. Receiver operating characteristic (ROC) curves of the trained six machine learning models for distinguishing PD patients from healthy controls (HCs) in the internal test cohort (**A**) and external test cohort (**B**). The confusion matrix of the SVM, MLP and KNN classification models predicted on the internal test cohort (**C**–**E**) and external test cohort (**F**–**H**). PD patients/HCs were treated as positive/negative, respectively. AUC, area under the ROC curve; SVM, support vector machine; MLP, multilayer perceptron; KNN, K-nearest neighbor

Figure 4 and Table 2 describe the classification performance of the six ML models, which were trained using the combined radiomics features in the internal and external test groups. The internal test cohort revealed that the six ML models achieved good performance with an AUC of 0.96–0.98, with the accuracy ranging from 0.80 to 0.90. Specifically, the SVM and MLP models both achieved the highest accuracy of 0.90 with an AUC of 0.97 (95% confidence interval (CI): 0.94–0.98). The RF model exhibited the lowest accuracy of 0.80, yet maintained a commendable AUC of 0.96 (95% CI: 0.94–0.98). The KNN, GNB, and AB models yielded intermediate accuracy of 0.86, 0.85, and 0.85, respectively, with an AUC of 0.98, 0.96, and 0.96, respectively. Using the AUC of MLP model as the reference, the DeLong test indicated no statistically significant differences in AUC values between the MLP model and all other evaluated models (all *p* > 0.05).

**Table 2:** Classification performance of trained six machine learning models for Parkinson’s disease (PD) patients in the internal and external test cohorts

		KNN	SVM	RF	GNB	AB	MLP
Accuracy	Internal test	*0.86*	0.90	0.80	0.85	0.85	0.90
	External test	0.79	*0.78*	0.70	0.77	0.77	*0.78*
Sensitive	Internal test	0.72	0.82	0.59	0.73	0.73	*0.81*
	External test	0.92	*0.86*	0.65	0.73	0.73	0.85
Specificity	Internal test	0.97	*0.96*	0.94	0.94	0.94	*0.96*
	External test	0.61	0.69	*0.76*	0.84	0.84	0.67
Recall	Internal test	0.72	0.82	0.59	0.73	0.73	*0.81*
	External test	0.92	*0.86*	0.65	0.73	0.73	0.85
Precision	Internal test	0.93	0.93	0.88	*0.89*	*0.89*	0.93
	External test	0.76	0.79	*0.80*	0.87	0.87	0.78
PPV	Internal test	0.93	0.93	0.88	*0.89*	*0.89*	0.93
	External test	0.76	0.79	*0.80*	0.87	0.87	0.78
NPV	Internal test	*0.83*	0.88	0.77	*0.83*	*0.83*	0.88
	External test	0.85	0.76	0.60	0.68	0.68	*0.77*
F1 score	Internal test	0.81	0.87	0.70	0.80	0.80	*0.86*
	External test	0.83	*0.82*	0.72	0.79	0.79	0.81
*p*-value^a^	Internal test	0.115	0.372	0.531	0.254	0.337	Reference
	External test	0.128	0.495	**< 0.005**	**< 0.005**	**< 0.005**	Reference

Underlined: optimal, italics: suboptimal, bold: statistically significant difference

*KNN* K-nearest neighbor, *RF* random forest, *SVM* support vector machine, *GNB* Gaussian Naive Bayes, *AB* adaptive boosting, *MLP* multilayer perceptron, *PPV* positive predictive value, *NPV* negative predictive value

^a^ DeLong test was performed to compare the statistical differences in AUCs between the MLP model and other models

### External classification performance

In the external test cohort, SVM, MLP and KNN models demonstrated slightly outperform the other models with AUC values over 0.80. The MLP model demonstrated the highest AUC of 0.85 (95% CI: 0.80–0.89) with an accuracy of 0.78. The KNN and SVM models emerged with the suboptimal AUC of 0.82 (95% CI: 0.77–0.87) and 0.81 (95% CI: 0.75–0.86), respectively. The accuracy was 0.79 and 0.78, respectively, which was comparable to that of the MLP model. The RF model, with an accuracy of 0.70, exhibited a lower AUC, which reached 0.77 (95% CI: 0.70–0.82). The GNB and AB models demonstrated the lowest AUC of 0.73 (95% CI: 0.67–0.79) for PD diagnosis, and the accuracy was both 0.77. DeLong test revealed no statistically significant differences in AUC values between the KNN and SVM models compared to the MLP model (*p* = 0.128 and *p* = 0.495, respectively). In contrast, the MLP model demonstrated significantly higher AUC values than the GNB, AB, and RF models (all *p* < 0.005).

## Discussion

In this study, we extracted the radiomics features from four key influenced deep nuclei affected in PD, including SN, RN, GP and PU regions based on the conventional T2W FLAIR images. Utilizing a cohort of 1727 subjects across five centers, we established six ML models using the combined radiomics features for discriminating PD patients from HCs. Our results showed that the trained ML models could classify PD and HC with commendable accuracy and AUC in both internal and external test cohorts.

The delineation of the four ROIs in our study was underpinned by their established correlation with the motor deficits characteristic of PD. Finally, a total of 20 radiomics features were selected. Specifically, there were five, two, three, and ten radiomics retained from regions of SN, RN, GP, and PU, respectively. The selection of 20 radiomics features, all higher-order, suggested that the subtle changes in first-order features such as signal intensity, shape, and volume may be challenging to discern visually, thus limiting the diagnostic capabilities of conventional imaging. SN is a nuclear complex, which contains the most substantial concentration of dopamine neurons in the brain. The progressive degeneration of dopamine neurons in the SN and the resultant striatal dopamine depletion are pathognomonic of PD and are primarily responsible for its motor symptoms [24]. In our work, the features extracted from SN illustrated the highest importance value, indicating the crucial role of SN in PD diagnosis. Numerous MRI techniques have accessed the nigral pathology in PD, including volume reduction [25], absence of dorsolateral nigral hyperintensity [26, 27], and loss of ‘swallow tail’ appearance [28]. However, the nigral pathology is common to all types of degenerative parkinsonism, suggesting that independent SN features may result in a low specificity of classification model. RN connects the cerebellum with the cerebral cortex. SN and RN are subcortical centers in charge of motor coordination [29]. Unexpectedly, the volume of RN increased in PD patients compared to the HCs, indicating that it may play a compensatory role in PD, especially in the pre-clinical stage, which may potentially ameliorate the dopamine depletion associated with the disease [30, 31]. However, the small volume may limit the detection of its subtle change in the disease progression. PU and GP are integral components of the striatum and recipients of dopamine from the SN, which are closely associated with motor disability in PD. In this work, PU contributed a significant number of radiomics features, highlighting the importance in disease differentiation. The early-stage dopamine loss in PD predominantly affects the posterior PU, with anterior PU involvement as the disease advances [32, 33]. PU, particularly its caudal portions, is supposed to be the most appropriate site for autograft of the dopamine-producing adrenal medullary tissue in PD [32]. Although controversial, some scholars have reported that both the volume and shape of PU and GP alter in PD [34–39]. A progressive atrophy accumulation in PU could be detected in the course of the disease, whereas the volume of GP may reduce only in advanced PD [37]. In addition, the GP internus is considered a commonly used target for deep brain stimulation, which is strongly recommended as an effective therapeutic intervention to alleviate the motor symptoms of PD [40]. Benefit from the adequate subjects enrolled, we fused the information of these 20 radiomics features for modeling, which would improve the accuracy of classification.

In this study, we applied six ML algorithms for PD classification. Those classic ML algorithms have good interpretability and are widely used in the binary classification problem of PD diagnosis. All models achieved good classification results with an AUC of 0.96–0.98 in the internal test set. In the external test cohorts, the AUC of SVM, MLP and KNN models exceeded 0.80, demonstrating better generalization than RF, GNB and AB models. The SVM model is known for its robust performance and low computational cost. It has been extensively utilized as a single algorithm on the ML studies for PD classification using various neuroimaging modalities, including three-dimensional structural T1-weighted MRI [16, 41], susceptibility-weighted imaging [15], quantitative susceptibility mapping [42], diffusion MRI [43], SPECT [44] and ^18^F-FDG PET [45]. They demonstrated an ideal performance with an accuracy range of 0.86 to 0.90. In our study, MLP and KNN showed comparable performance with SVM in the PD diagnosis. MLP, also known as artificial neural network, is a popular feed-forward neural network algorithm. Chakraborty et al [46] extracted the textural, morphological and statistical features from eight subcortical structures on 3.0-T T1W MRI scans, and then the best-performing features were trained using four ML algorithms, among which MLP performed best, with an overall accuracy of 95.3%. KNN is a simple ML model, whereas it showed better classification results than GNB and AB models, suggesting that the simple ML algorithm may also offer promising classification outcomes for binary problem. One meta-analysis showed that the pooled AUC of radiomics-based ML models for PD diagnosis was 0.871 (95% CI: 0.853–0.890) in the validation set [47]. The AUC of the internal test cohort in our work is obviously superior to the pooled AUC. In general, the trained ML models based on the conventional T2W FLAIR images performed with moderate to good accuracy in the internal and external test cohorts.

To our knowledge, few studies have applied radiomics for PD diagnosis based on the conventional MRI. Liu et al [17] used conventional T2W images from 69 PD patients and 69 HCs to establish the classification model of PD. They built caudate nucleus and PU radiomics signatures, respectively. In the training group, the AUC of caudate nucleus and PU was 0.9410 and 0.8767, respectively. In the test group, the AUC was 0.7732 and 0.7143, respectively. In our study, we extracted and combined the radiomics features of four key affected regions in PD, improving the sensitivity and specificity of the models. We included the largest cohort of 1727 subjects to date, and the images were obtained across various MRI scanners with different manufacturers and field strength. The substantial size of our training and test datasets contributed significantly to mitigating overfitting, thereby enhancing the models’ robustness, transferability, and generalization capabilities.

### Limitations

There are still some limitations in our study. First, this was a retrospective study. Prospective validation is needed to evaluate the reproducibility and the potential for clinical translation of the models. Second, while our ML models demonstrated high accuracy for PD diagnosis, integrating it with accessible biological indicators such as the Unified Parkinson’s Disease Rating Scale score could further augment diagnostic precision. Third, manual delineation by radiologists was employed in our work in order to ensure the correct segmentation of ROIs, however, it is burdensome and resource-intensive. At present, deep learning methods have achieved promising results in deep nuclei segmentation with an accuracy of 80–92% [23, 48–50]. We have also carried out related work on the automatic segmentation of deep nuclei. Further development of an automated segmentation model could streamline the diagnostic pipeline. Lastly, the diagnosis of PD was based on clinical criteria instead of neuropathological results. The clinical PD classification models need further neuropathological validation.

## Conclusion

In summary, our ML models, grounded in conventional T2W FLAIR images, have shown promising results in discriminating PD patients from HCs. We provided an objective, semi-automated, and efficacious method for PD screening in the early stage. Future validation in the prospective data will be instrumental in solidifying the reliability of these models.

## Supplementary information

ELECTRONIC SUPPLEMENTARY MATERIAL

## Data Availability

Data supporting the findings and code used are available from the corresponding author (D.Y.G.) on request. All software used in this submission is publicly accessible through the links provided at citation.

## Abbreviations

AB: Adaptive boosting
AUC: Area under the curve
DSC: Dice similarity coefficient
FLAIR: Fluid-attenuated inversion recovery
GNB: Gaussian Naive Bayes
GP: Globus pallidus
HC: Healthy control
KNN: K-nearest neighbor
LASSO: Least Absolute Shrinkage and Selection Operator
ML: Machine learning
MLP: Multilayer perceptron
NPV: Negative predictive value
PD: Parkinson’s disease
PPMI: The Parkinson’s Progression Marker Initiative
PU: Putamen
RF: Random forest
RN: Red nucleus
ROC: Receiver operating characteristic
ROI: Region of interest
SN: Substantia nigra
SVM: Support vector machine
