# Supplementary material for: Harnessing routine MRI for the early screening of Parkinson’s disease: a multicenter machine learning study using T2-weighted FLAIR imaging
Source: Insights Imaging. 2025 Apr 26;16:92. doi: 10.1186/s13244-025-01961-3 (PMC12033128; doi:10.1186/s13244-025-01961-3)
Supplement: Supplementary file 1 — ELECTRONIC SUPPLEMENTARY MATERIAL [file 13244_2025_1961_MOESM1_ESM.pdf]

Harnessing Routine MRI for the Early Screening of Parkinson's Disease: A Multicenter Machine Learning Study Using T2-Weighted FLAIR Imaging

ELECTRONIC SUPPLEMENTARY MATERIAL

Supplementary Table 1 MRI Acquisition protocol for each cohort.

MRI scanner	TR /ms	TI /ms	TE /ms	Matrix /pixels	Pixel spacing /mm	Thickness /mm	Interval /mm	Slices	FA /degree	Field Strength /tesla	Sources
Veria, Simense	7000	2250	97	384×512	0.47×0.47	5	6	24	150	3.0	HS
Prisma, Simense	7500	2299	86	176×256	0.94×0.94	6	7.8	18	150	3.0	HS
Discovery 750, GE	8525	2100	140	512×512	0.47×0.47	6	8	18	111	3.0	HS
uMR 790, UIH	8000	2440	102	276×384	0.65×0.65	6	7.8	18	150	3.0	HS
Vantage Tian, Canon	7000	2150	120	256×256	0.47×0.47	6	8	16	90	3.0	HS
uMR 670, UIH	8000	2300	128	256×167	0.45×0.45	6	7.2	20	150	1.5	NB
MAGNETOM Vida, Siemens	7000	2216	96	320×240	0.72×0.72	5	6.5	23	120	3.0	NB
Discovery 750w, GE	9000	2473	120	256×256	0.47×0.47	5	6.5	20	160	3.0	NT
Discovery 750w, GE	9000	2473	90	320×192	0.47×0.47	5	6.5	20	160	3.0	XZ
Symphony, Siemens	10000	2500	120	256×256	0.90×0.90	5	/	25	180	1.5	PPMI
Achieva, Philips	11000	2800	125	560×560	0.40×0.40	4	/	28	90	3.0	PPMI

Abbreviations: MRI, magnetic resonance imaging; TR, repetition time; TI, inversion time; TE, time of echo; FA, flip angle; HS, Huashan hospital affiliated to Fudan university; NB, Ningbo first hospital; NT, affiliated hospital and medical school of Nantong university; XZ, the second affiliated hospital of Xuzhou medical university; PPMI, the Parkinson's progression marker initiative.

Insights Imaging (2025) Fu J, Chen H, Xu C, et al.

Supplementary Table 2 Calculation formulas of evaluation metrics of models

$\mathrm{ACC} = (TP+TN)/(TP+TN+FP+FN)$	(1)
$\mathrm{SEN/Recall} = TP\,/\,(TP + FN)$	(2)
$\mathrm{SPE} = TN\,/\,(TN + FP)$	(3)
PPV/Precision = TP/(TP+FP)	(4)
NPV = TN/(TN+FN)	(5)
$F1\,Score = \frac{2*(TP\,/\,(TP + FP))(TP\,/\,(TP + FN))}{(TP\,/\,(TP + FP) + TP\,/\,(TP + FN))}$	(6)

Note: ACC = Accuracy, TP=true positive, TN=true negative, FP=false positive, FN=false negative, SEN = sensitivity, SPE = specificity, PPV = positive predictive value, NPV = negative predictive value.

Supplementary Table 3 Demographic information for each dataset in the external test cohort

**Dataset**		**Subjects**		**Age (y)**			**Sex (M/F)**		
	HC	PD	HC	PD	P-value	HC	PD	P-value
NT	94	97	65.44(7.74)	66.33(7.62)	0.422	43/51	55/42	0.149
XZ	31	35	62.52(10.99)	65.80(6.42)	0.152	15/16	45620	0.101
NB	36	39	58.86(4.83)	62.00(9.30)	0.074	13/23	18/21	0.384
PPMI	59	124	61.36(12.85)	59.51(10.36)	0.299	36/23	71/53	0.632
Total	220	295	62.85(9.77)	61.82(9.47)	0.974	107/113	168/127	0.062

Abbreviations: y, years; M, male; F, female; HC, healthy control; PD, Parkinson’s disease; NB, Ningbo first hospital; NT, affiliated hospital and medical school of Nantong university; XZ, the second affiliated hospital of Xuzhou medical university; PPMI, the Parkinson’s Progression Marker Initiative. Data are represented as mean (standard deviation) where appropriate.
